# **Vascular and lymphatic complications after renal transplant**

**DOI:** 10.1007/s00261-025-05032-5

**Published:** 2025-06-06

**Authors:** Claire E. White-Dzuro, Demetrios J. Geanon, Brian M. Fagel, Lena Khanolkar, Shelby K. Frantz, Christopher M. Baron, Reza A. Imani-Shikhabadi, Nicholas Voutsinas

**Affiliations:** 1 Vanderbilt University School of Medicine, Nashville, USA; 2 Vanderbilt University Medical Center, Nashville, USA

**Keywords:** Renal transplant, Vascular complication, Lymphatic complication, Endovascular management, Interventional radiology

## Abstract

Renal transplant is a life-saving treatment option for patients with end-stage renal disease. As with any intervention, transplantation is not without potential complications, which include disruption to arterial, venous and lymphatic structures in the region and can involve either native or transplanted anatomy. Management options range from open surgical intervention to endovascular procedures, the latter of which have become increasingly more prevalent due to their minimally invasive nature. Interventional Radiology has a diverse procedural skillset that can be utilized for successful management of post-transplant complications. Treatment modalities include, but are not limited to, embolization, thrombectomy and stent placement. The goal of this article is to explore common vascular and lymphatic complications that occur following renal transplant and review relevant minimally invasive management options. Positive treatment outcomes are essential to ensure graft, and in turn, patient survival.

## Introduction

Renal transplantation is the preferred treatment for patients living with end-stage renal disease (ESRD), defined as a GFR < 15 mL/min. The most common etiologies of ESRD requiring eventual transplant include diabetes mellitus and hypertension [1]. Renal transplant has been demonstrated to be a successful, life-saving procedure with a one-year graft survival rate greater than 94%.^2^ While donor kidneys can be obtained from a living or cadaveric donor, studies have demonstrated longer graft and patient survival in patients who undergo transplantation from living as opposed to deceased donors [1]. In 2023, five-year graft survival was reported at 80–90% after living-donor transplant compared to 66–82% after deceased-donor transplant [1]. Studies have also demonstrated an increase in graft half-life for both living and deceased donor transplantation over the past thirty years, indicating an improvement in the long-term success of the procedure [2]. This advancement is hypothesized to be the result of a variety of improvements including advanced procurement and preservation techniques as well as more sophisticated management of transplant complications and recipient comorbidities [2]. Moreover, xenotransplantation, the insertion of cells, tissue or organs from an animal source into a human host, represents a promising advancement that could eliminate or at the very least, reduce the significant organ supply shortage that currently exists [3]. To date, four cases of renal xenotransplant have been successfully completed.

Both donor and recipient require adequate imaging work-up prior to transplant surgery. Any potential donor should undergo CT angiography with and without contrast. The non-contrasted images can help evaluate the extent of atherosclerotic disease within the transplant vasculature and identify possible nephrolithiasis. The contrasted images can help assess vascular anatomy through the arterial and venous phases as well as the ureters during the delayed excretory phase. Transplant recipients should get a non-contrasted CT abdomen and pelvis. This allows the surgeon to evaluate pelvic anatomy and assess the atherosclerotic disease burden of the iliac vessels to determine the optimal site of re-implantation. Occasionally, CT angiography or venography may be required for further evaluation, although this should be weighed against the risk of contrast administration.

Kidney transplant surgery involves three important components: donor harvest, back table preparation and recipient implantation [4]. Donor harvest surgery can be performed in an open, laparoscopic or robot-assisted fashion, with minimally invasive options more commonly utilized due to reductions in post-operative pain with equivalent clinical outcomes [5, 6]. During the procedure, the kidney is mobilized from surrounding retroperitoneal structures. The distal ureter is ligated followed by the renal vessels and the kidney is removed. The harvested kidney is then placed on ice on a back table to minimize metabolic demands and resultant ischemia [4]. There, the kidney is inspected and prepared for re-implantation [6]. In the recipient, open dissection is performed in either the right or left lower quadrant of the abdomen to the location of re-implantation. The transplanted renal artery and vein are anastomosed to the iliac vessels, most commonly the external iliac artery and vein, and the transplanted ureter is anastomosed to the bladder. As with any surgical procedure, especially one involving both a donor and recipient patient and several complex steps, complications can occur. The goal of this article is to explore common vascular and lymphatic complications that occur after renal transplant to both transplanted and native anatomy and review relevant endovascular management options.

## Renal transplant vascular anatomy

**Fig. 1:**
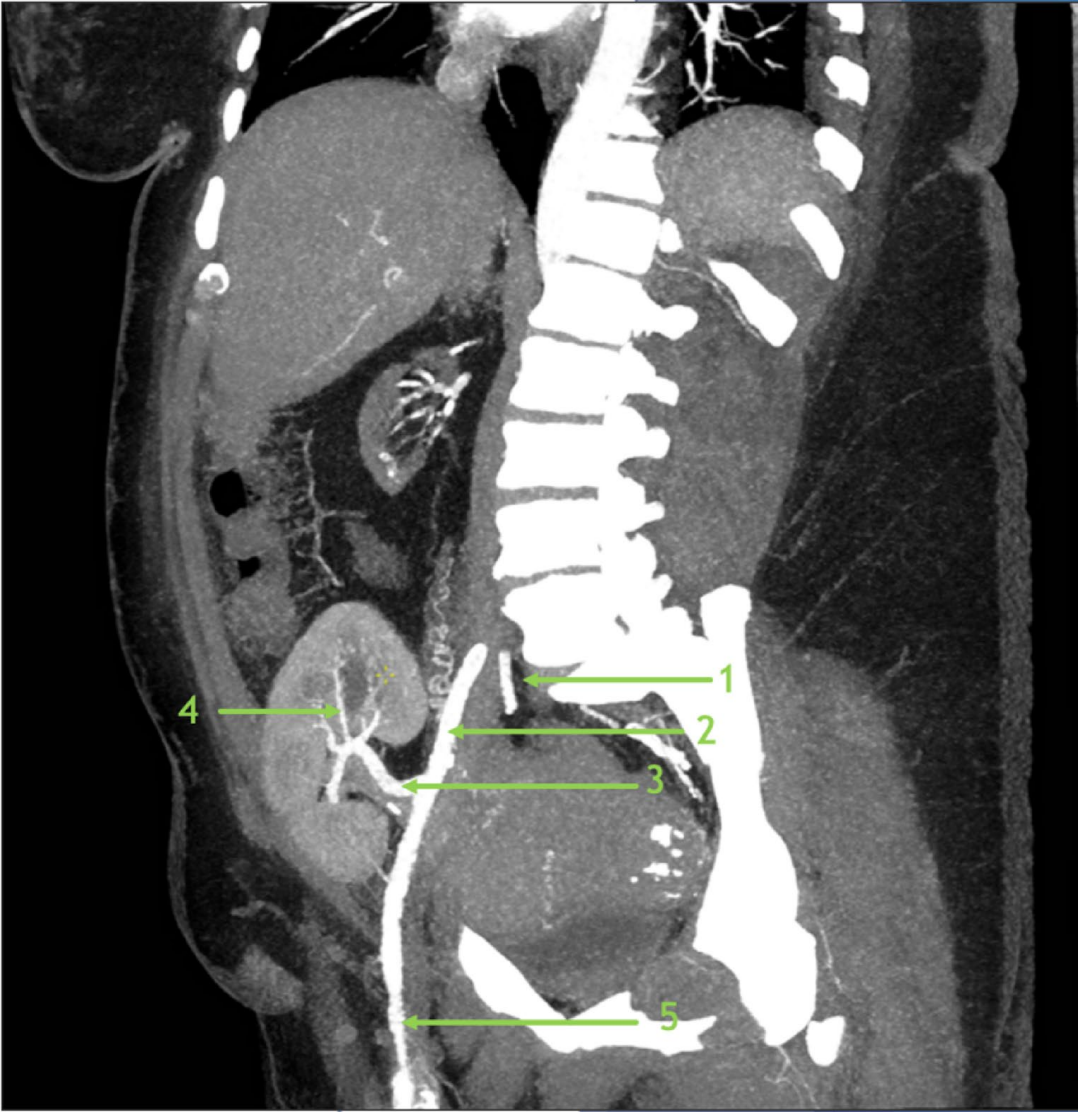
Sagittal CT angiography demonstrating relevant arterial anatomy of a transplanted kidney. 1- Internal Iliac Artery; 2- External Iliac Artery; 3- Main Renal Transplant Artery; 4– Segmental Renal Transplant Artery; 5 - Common Femoral Artery

Kidney implantation can occur in the right or left extraperitoneal pelvis, with anastomosis most commonly to the external iliac vessels (Fig. 1). Prior to surgery, living donors and recipients will undergo imaging to assess atherosclerotic burden in the iliac vasculature and determine the optimal location for implantation. Patients may undergo preoperative external iliac revascularization if the vessels do not demonstrate adequate size or blood flow [7]. However, if the vessels remain a suboptimal vascular source, studies have validated the internal iliac vessels as a reliable anastomotic site [8–10].

## Arterial complications

Arterial complications are the most common type of vascular complication after renal transplant. Early recognition and treatment are crucial in order to restore adequate blood flow to the organ and prevent graft failure. In this section, we will: outline common post-operative complications to both transplanted and native arteries including stenosis, thrombosis and dissection; define pertinent clinical and imaging findings; and discuss optimal endovascular treatment options (Table 1).

**Table 1:** Summary of imaging findings and treatment of Post-Transplant arterial complications

Arterial Complications	Imaging Findings	Endovascular Treatment
Transplant Artery		
*Stenosis*	*US*: Increased peak systolic velocities in the transplant artery; tardus parvus waveforms *CTA/MRA*: Focal decrease in diameter of the transplanted artery *Angiography (CO2 or contrast)*: Stenosis of renal artery; blunted systolic upstrokes on invasive hemodynamic monitoring	Angioplasty +/- stent placement
*Thrombosis*	*US*: Lack of both arterial and venous flow in the graft *Contrasted CT*: Swollen graft with decreased enhancement +/- cortical rim enhancement or subcapsular fluid *CTA*: Hypodensity within transplant artery lumen *Angiography*: Failed opacification of transplant artery	Intra-arterial thrombolysis or thrombectomy
Native artery		
*Stenosis*	*US*: Increased peak velocity within the stenotic region; loss of typical triphasic signal distal to stenosis *CTA/MRA/Angiography*: Focal narrowing of the artery	Angioplasty +/- stent placement
*Dissection*	*US*: Possible signs of stenosis *CT/MR*: Dissection Flap *Angiography*: Dissection flap; irregularity of the artery wall	Stent placement
*Thrombosis*	*US*: Lack of arterial flow in the thrombosed portion *CTA*: Hypodensity within artery lumen *Angiography*: Failed opacification of artery	Thrombectomy +/- stent placement
Biopsy-related		
*Hemorrhage*	*US*: Heterogeneously echogenic collection *CT*: Acute - hyperattenuating free fluid; Chronic– hypoattenuating free fluid *Angiography*: Active contrast extravasation	Drainage or trans-catheter embolization if failure to control with conservative measures
*AVF*	*US*: High-velocity connection between artery and vein; color overlying surrounding soft tissue; decreased arterial resistance, venous pulsatility, venous dilation *Angiography*: Early venous opacification	Trans-catheter embolization
*Pseudoaneurysm*	*US*: Cystic-appearing mass with blood flow entering and leaving *CT*: Focal high attenuating lesion with similar density to surrounding arteries *Angiography*: Focal dilation of a segmental artery	Trans-catheter embolization

### Transplant artery stenosis

Renal transplant artery stenosis is the most common vascular complication. Although previously believed to occur in up to 23% of all transplanted kidneys, recent large cohort studies have demonstrated a much lower overall incidence of approximately 1–3% [11, 12]. Renal transplant artery stenosis is defined as a > 50% decrease in intraluminal diameter or > 15 mmHg decrease in pressure across a stenotic segment [13]. Potential etiologies include faulty surgical technique with damage to the donor artery during harvesting, kinking and compression of the donor artery during creation of the anastomosis, donor-recipient arterial size discrepancy and chronic rejection [14–16]. Development of transplant renal artery stenosis is also associated with CMV infection [17]. Recognition and treatment are important as stenosis can lead to clinically significant graft dysfunction.

While timing is variable depending on etiology, transplant renal artery stenosis typically arises within the first year post-operatively, with most cases developing within the first 3–6 months [18]. Symptoms that warrant work-up for renal transplant artery stenosis include new or worsening hypertension, fluid overload or renal dysfunction [19–21]. Some patients may present with Pickering syndrome, defined as a hypertensive crisis and flash pulmonary edema secondary to either bilateral renal artery stenosis in patients with two functioning kidneys or unilateral stenosis in a patient with a single/transplanted kidney [22, 23].

**Fig. 2:**
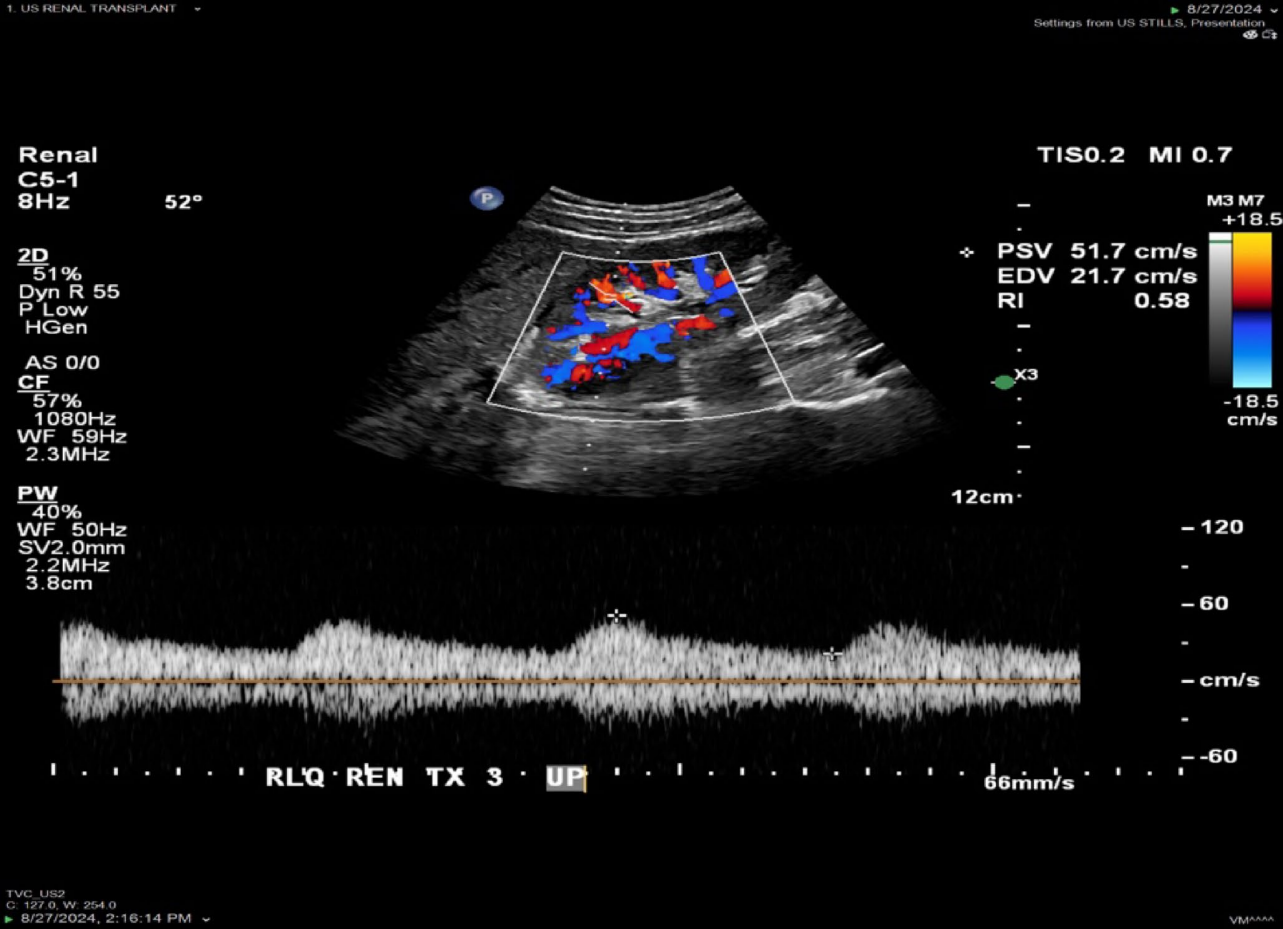
31-year-old female status post RLQ renal transplantation with worsening renal function. Doppler US demonstrated tardus parvus waveforms and decreased resistive index (0.58) in the upper pole segmental renal arteries, suspicious for upstream renal artery stenosis

**Fig. 3:**
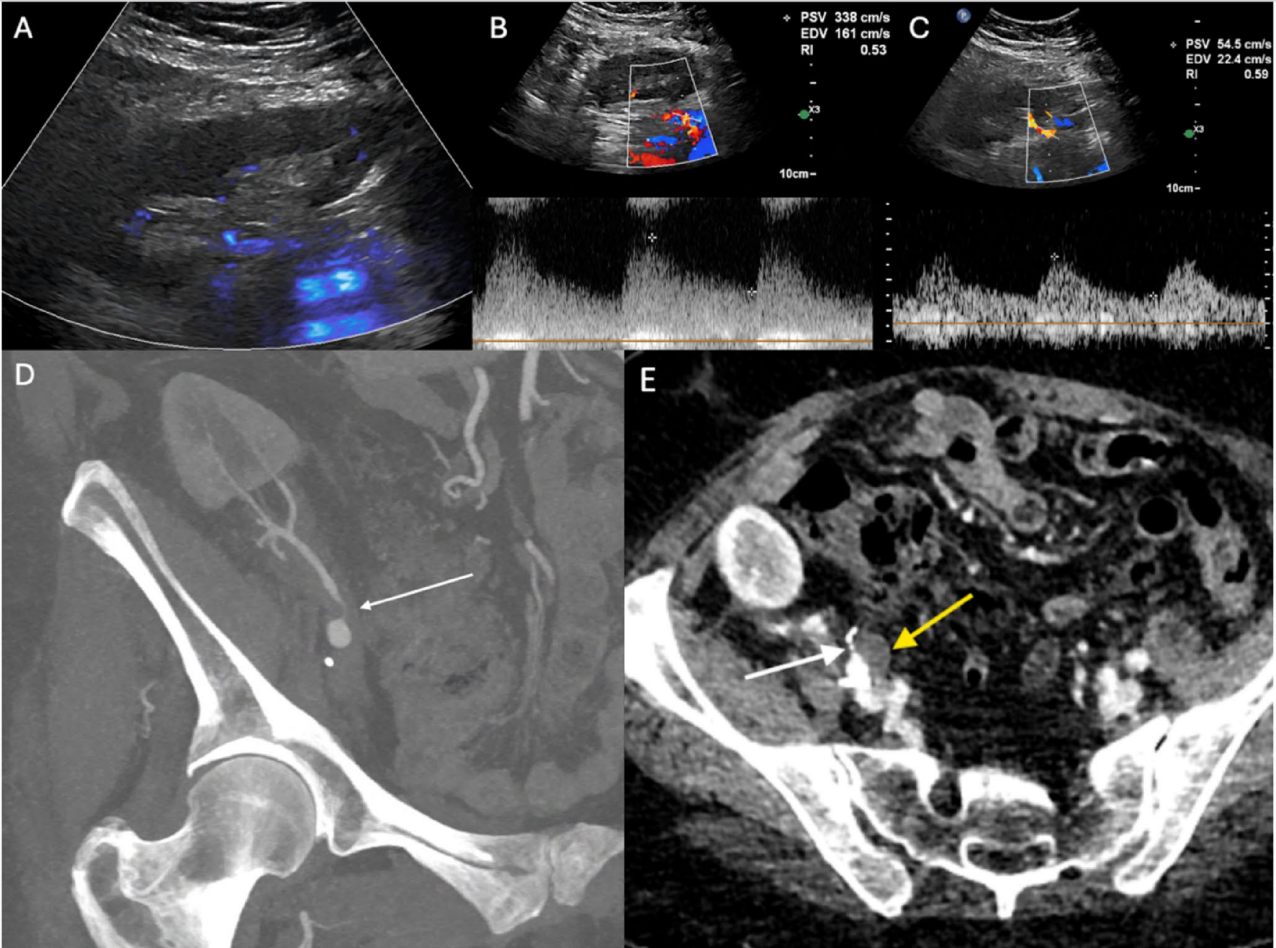
67-year-old female status post RLQ renal transplant for ESRD, complicated by donor urine cultures positive for candida albicans. Doppler US demonstrated homogenously decreased parenchymal flow throughout the graft on Power Doppler evaluation (A). An elevated peak systolic velocity of 338 cm/s in the proximal renal artery near the anastomosis was seen on Spectral Doppler evaluation (B). Doppler US of the mid renal artery demonstrated tardus parvus waveforms with a decreased peak systolic velocity of 55 cm/s and decreased resistive index of 0.59 (C). Coronal reformatted CT angiography demonstrated a short segment, severe proximal renal artery stenosis immediately distal to the anastomosis (white arrow) (D). Sagittal reformatted CT angiography revealed a hypoattenuating focus exerting mass effect on the proximal renal artery resulting in severe stenosis (E). Patient was taken to the operating room for further evaluation. A small serous collection (yellow arrow) near the arterial anastomotic site was identified causing the stenosis (white arrow). End-to-side re-anastomosis between the renal transplant artery and external iliac artery was performed

**Fig. 4:**
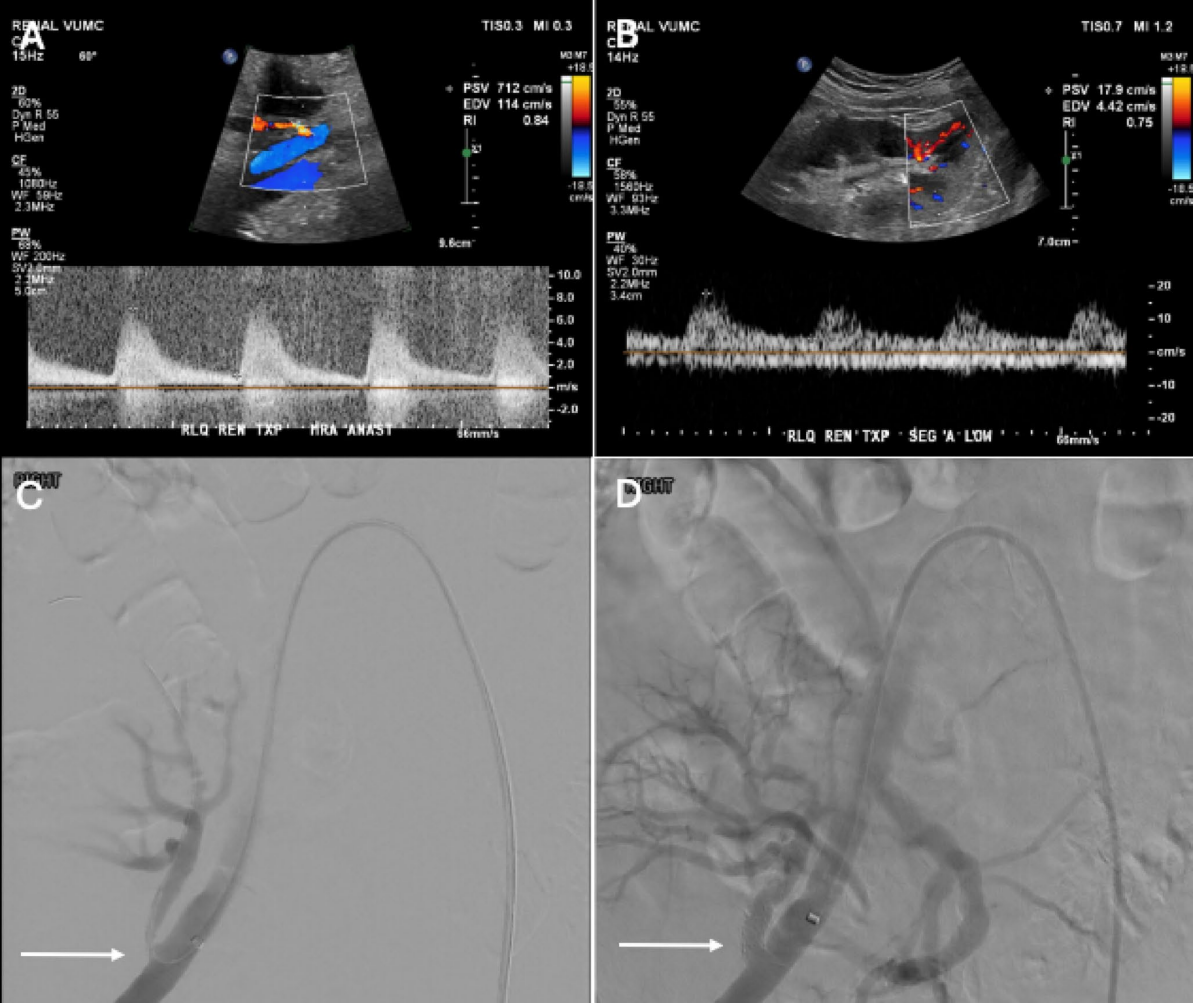
57-year-old male status post RLQ renal transplant admitted 3 months post-operatively for acute renal failure and volume overload. Spectral Doppler US revealed significantly elevated peak systolic velocities in various segments of the main renal artery (anastomotic site– 712 cm/s) (A). Intrarenal segmental branches on Spectral Doppler US demonstrated tardus parvus waveforms (B). Right external iliac angiogram confirmed stenosis of the proximal transplant renal artery (C). A 6 × 14 mm Express stent (Boston Scientific) was deployed across the stenosis. Post-deployment angiography demonstrated complete resolution of the stenosis with markedly improved blood flow to the transplanted kidney (D)

**Fig. 5:**
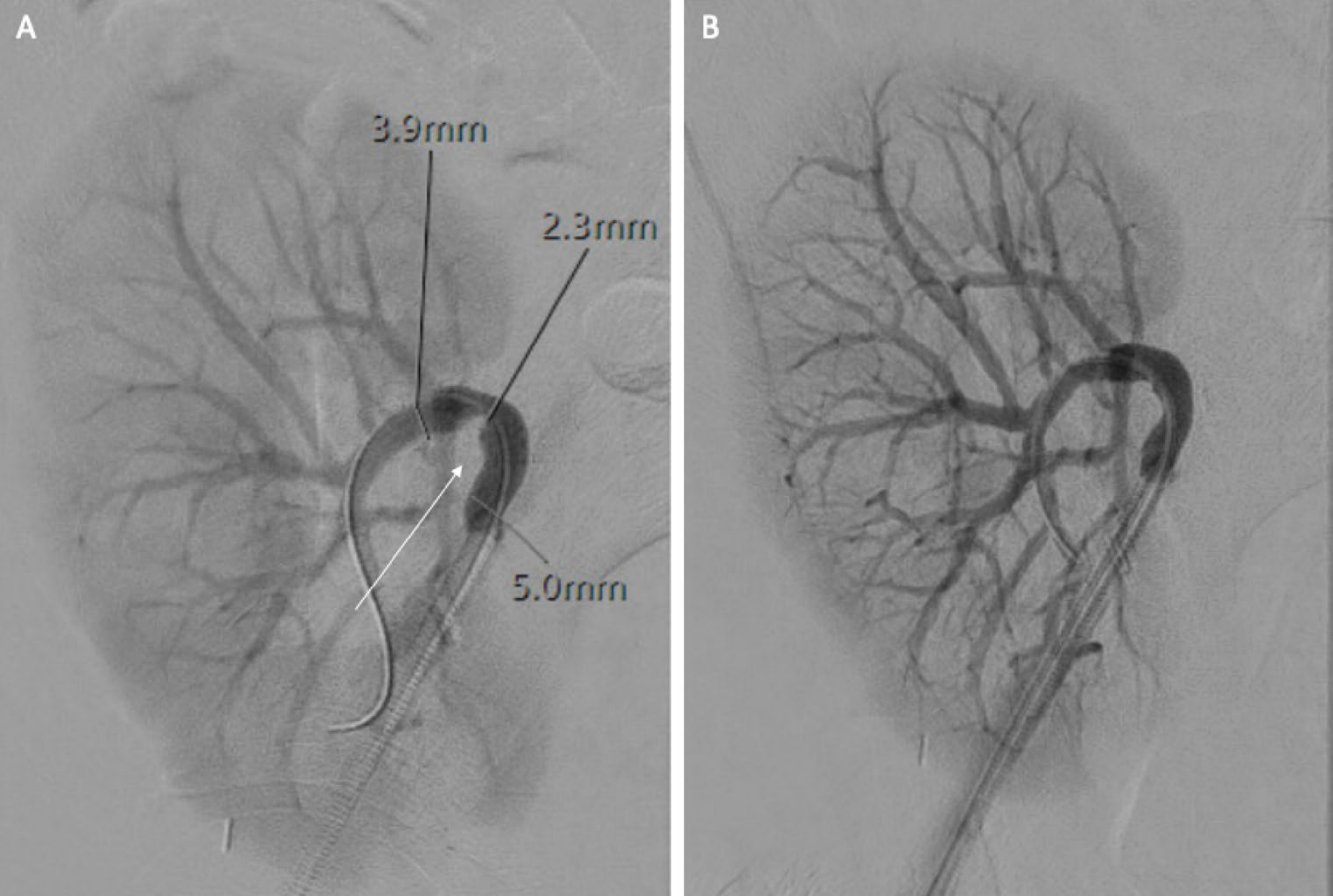
57-year-old female with RLQ renal transplant two years prior, found to have elevated peak systolic velocities up to 301 cm/s in the transplanted main renal artery, concerning for renal artery stenosis (not shown). Initial angiogram demonstrated a small region of stenosis in the main renal artery (A). Systolic pressures were obtained distal and proximal to the area of stenosis (distal: 102/45 (66) mmHg; proximal: 107/46 (67) mmHg). The area of stenosis was no longer visualized on repeat angiography (B). Given the lack of pressure gradient and resolution on repeat angiography, the stenosis was believed to be an area of focal vasospasm, and no intervention was performed. The patient was managed medically

**Fig. 6:**
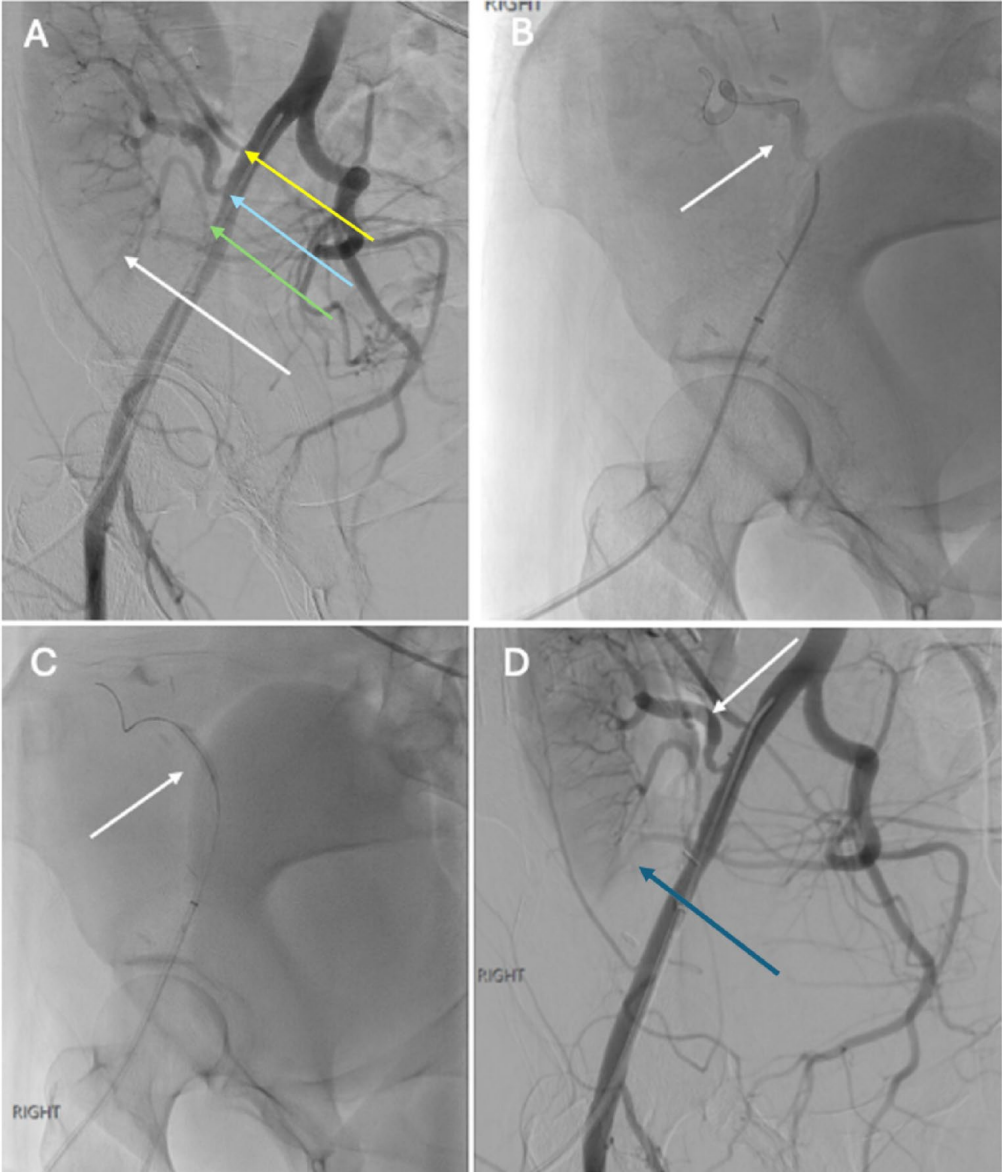
34-year-old female status post RLQ renal transplant complicated by renal artery stenosis seen on US and CT imaging (not shown). Right external iliac angiogram demonstrated three transplant renal arteries (superior– yellow arrow; middle– blue arrow; inferior– green arrow) with hypoperfusion to the mid and lower pole (white arrow), which corresponded to prior CT findings (A). Additional angiography demonstrated tortuosity and stenosis of the transplant middle renal artery proximally (B). Invasive hemodynamic monitoring demonstrated blunted systolic upstrokes (not shown), so angioplasty was performed with a 3.5 mm balloon (C). Subsequent angiography demonstrated improved patency of the middle renal artery (white arrow) and increased perfusion to the mid and lower pole renal transplant (blue arrow) (D)

Doppler US is the preferred initial imaging modality for diagnosis [24]. In patients with transplant artery stenosis, Doppler US will demonstrate increased peak systolic velocities (PSV) in the stenotic segment, increased velocity gradient between the stenotic and pre-stenotic segment and alteration of the waveform downstream of the stenosis (Figs. 2, 3 and 4). There is no universal criteria to define significant stenosis on Doppler US, although some authors suggest criteria of (1) PSV > 200 cm/s, (2) greater than 2:1 ratio of the stenotic to pre-stenotic PSV, and (3) marked downstream turbulence with tardus parvus waveforms or spectral broadening distal to the stenosis [25]. More strict criteria will allow for increased specificity. For example, at our institution, two of the following three criteria are used to diagnose renal artery stenosis: PSV > 300 cm/s, greater than 3:1 PSV ratio and downstream turbulence with tardus parvus or aliasing waveforms. If Doppler US findings are suspicious for renal artery stenosis, subsequent imaging with CT or MR angiography may be obtained prior to invasive catheter angiography (Fig. 3) [26, 27]. Renal MRA without contrast has been demonstrated to be a safe, reliable, non-invasive imaging modality to effectively assess stenosis, eliminating the need to subject patients with renal insufficiency to potentially harmful contrast agents [28]. Prior to definitive treatment, iodinated contrast or CO2 catheter angiography with or without invasive pressure measurements are necessary (Figs. 4, 5 and 6). CO2 can be especially useful in this population as it has no known nephrotoxicity [29]. Moreover, the anterior location of renal transplants is also beneficial as the gas rises into the graft vessels [30].

For renal artery stenosis, optimal treatment is dependent on the degree and location of the stenotic segment. Options include medical management, endovascular intervention or open-surgical management, including surgical re-implantation (Figs. 3, 4, 5 and 6) [18, 31]. Endovascular treatment options include angioplasty and stent placement (Figs. 4 and 6). These interventions have rates of clinical success, defined as improvement in either blood pressure or renal function, between 65% and 94% and a reported restenosis rate of 4.2% [32, 33].

### Transplant artery thrombosis

Transplant artery thrombosis is a relatively rare complication with a reported incidence of 0.08–1% [34–38]. While the cause is likely multifactorial, identified risk factors include hypercoagulable states, downstream renal artery stenosis, prolonged ischemia time and hypovolemia [39, 40]. Symptoms include sudden oliguria or anuria as well as pain near the graft site [41]. Similar to transplant renal artery stenosis, color Doppler US is the initial imaging modality to investigate potential thrombosis with contrasted CT or CT angiography used as appropriate confirmatory testing [24, 40]. In the setting of arterial thrombosis, doppler US demonstrates an absence of both arterial and venous flow [24]. Contrasted CT shows a swollen, patchy or non-enhancing graft with or without cortical rim enhancement or subcapsular fluid [42]. On CTA, a lack of opacity of the graft as well as a hypodensity within the lumen of the vessel is visualized [43]. Urgent treatment is important due to the risk of ischemic necrosis and graft failure. Common techniques to manage transplant artery thrombosis include intra-arterial thrombolysis or surgical thrombectomy [44].

### Complications of native artery

**Fig. 7:**
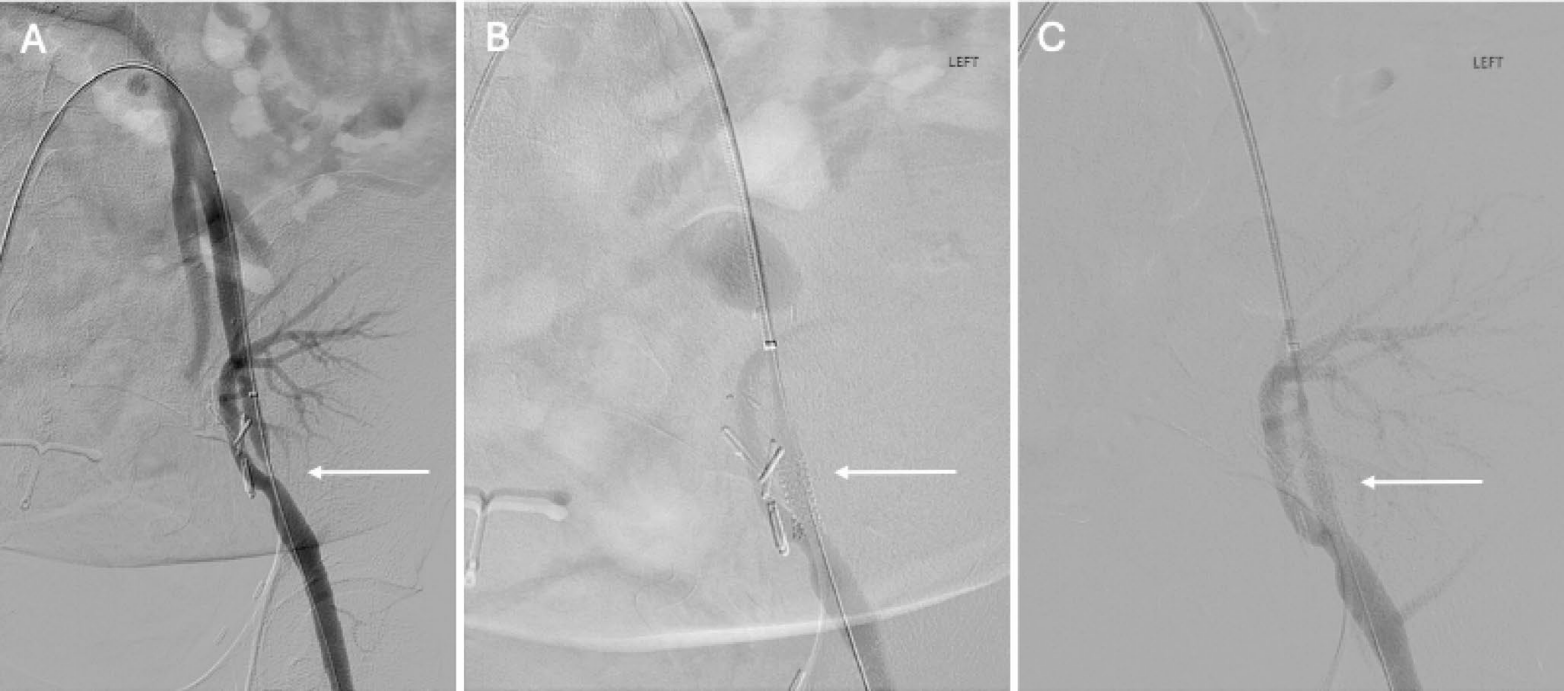
​​ Patient with history of LLQ renal transplantation CT angiography demonstrating severe stenosis of the native external iliac artery (not shown). Left external iliac angiogram demonstrated near-complete occlusion of the external iliac artery proximal to origin of the arterial anastomosis (A). Thestenosis was recanalized using a 0.035 Glidewire Advantage and a 5 Fr, 65 cm angled-tapered catheter. Ultimately, a 6 × 18 mm uncovered Express stent (Boston Scientific) was deployed across the region of stenosis (B). Post-procedure angiogram demonstrated no residual stenosis and appropriate flow into the renal artery and left lower extremity (C)

**Fig. 8:**
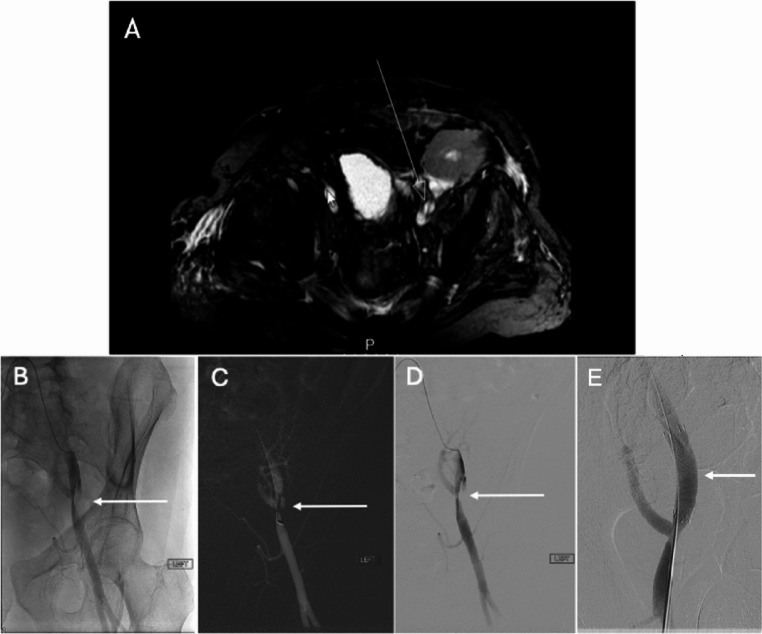
67-year-old male with combined LLQ renal and heart transplant presenting with acute renal failure. Doppler US (not shown) demonstrated elevated peak systolic velocities within the main renal artery up to 398 cm/s concerning for renal artery stenosis. Pelvic MRA demonstrated abnormal signal in the left external iliac artery near the anastomosis, concerning for dissection (A). Contrast (B, D) and CO2 (C) angiography confirmed a dissection flap within the mid external iliac artery extending above and below the level of the transplant renal artery origin with decreased flow to the renal transplant. A 10 × 40 mm balloon-expandable bare metal stent was deployed just proximal to the anastomotic site. Post-procedure angiography revealed appropriate stent positioning just proximal to the anastomosis with significantly increased flow to the renal transplant (E)

Surgical manipulation of the external iliac artery during graft transplantation can, rarely, lead to arterial compromise, causing complications such as stenosis, dissection and thrombosis of the native artery (Figs. 7 and 8). Due to the disruption of lower extremity blood flow, patients will present with symptoms not only of graft dysfunction but also signs of distal limb ischemia, such as claudication and exercise intolerance [45–47].

Iliac artery stenosis proximal to the transplanted artery has a reported incidence of 1.5% [48]. There are several proposed mechanisms leading to the development of iliac artery stenosis, which include progression of existing atherosclerotic disease and intimal hyperplasia resulting from clamp-related intra-operative trauma [46, 47]. Peripheral arterial disease is another risk factor, further suggesting that higher baseline atherosclerotic burden places patients at increased risk of complication [46]. Doppler US, followed by CT angiography, are appropriate imaging modalities if there is concern of large artery stenosis. Doppler US will show an increase in peak velocity within the stenotic region greater than 200 cm/s as well as a loss of typical triphasic signal downstream [48]. Management can involve open procedures, such as endarterectomy [49] and bypass around the stenotic or occluded site [50], to endovascular revascularization using balloon angioplasty with or without stent placement (Fig. 7) [47, 51, 52].

Arterial dissection is another incredibly rare but dangerous complication that can occur during renal transplant. Similar to native artery stenosis, it can develop because of vascular clamp-related injury or improper surgical technique [45]. Patients at risk of developing native artery dissection include those with significant atherosclerotic disease, hypertension and vascular collagen disorders [53–55]. Open management may involve endarterectomy with intimal tacking, or the use of bypass or interposition grafts, often with re-anastomosis of the transplanted kidney [53, 54]. Endovascular management primarily involves stenting of the native artery or transplanted artery occluded by the dissection (Fig. 8) [53, 54].

Thrombosis of the iliac artery, with or without thrombosis of the transplanted renal artery, has only been reported in a handful of case reports. It can occur due to pre-existing dissection, atherosclerotic occlusion or immune reaction such as a hyperacute rejection [56]. Endovascular management involves thrombectomy, commonly followed by stent placement [56, 57].

### Transplant biopsy-related complications

Transplant renal biopsy is the gold standard for assessing the etiology of graft dysfunction [58, 59]. The primary indication for biopsy is decreased graft function, especially in the setting of proteinuria or new hypertension [60]. In addition, some centers perform surveillance biopsies, or “protocol biopsies”, within the first year of transplant to evaluate for subclinical rejection. However, recent studies have demonstrated that performing protocol biopsies have no graft survival benefit and thus, advocate for implementing surveillance exclusively for those at high risk of rejection [61].

**Fig. 9:**
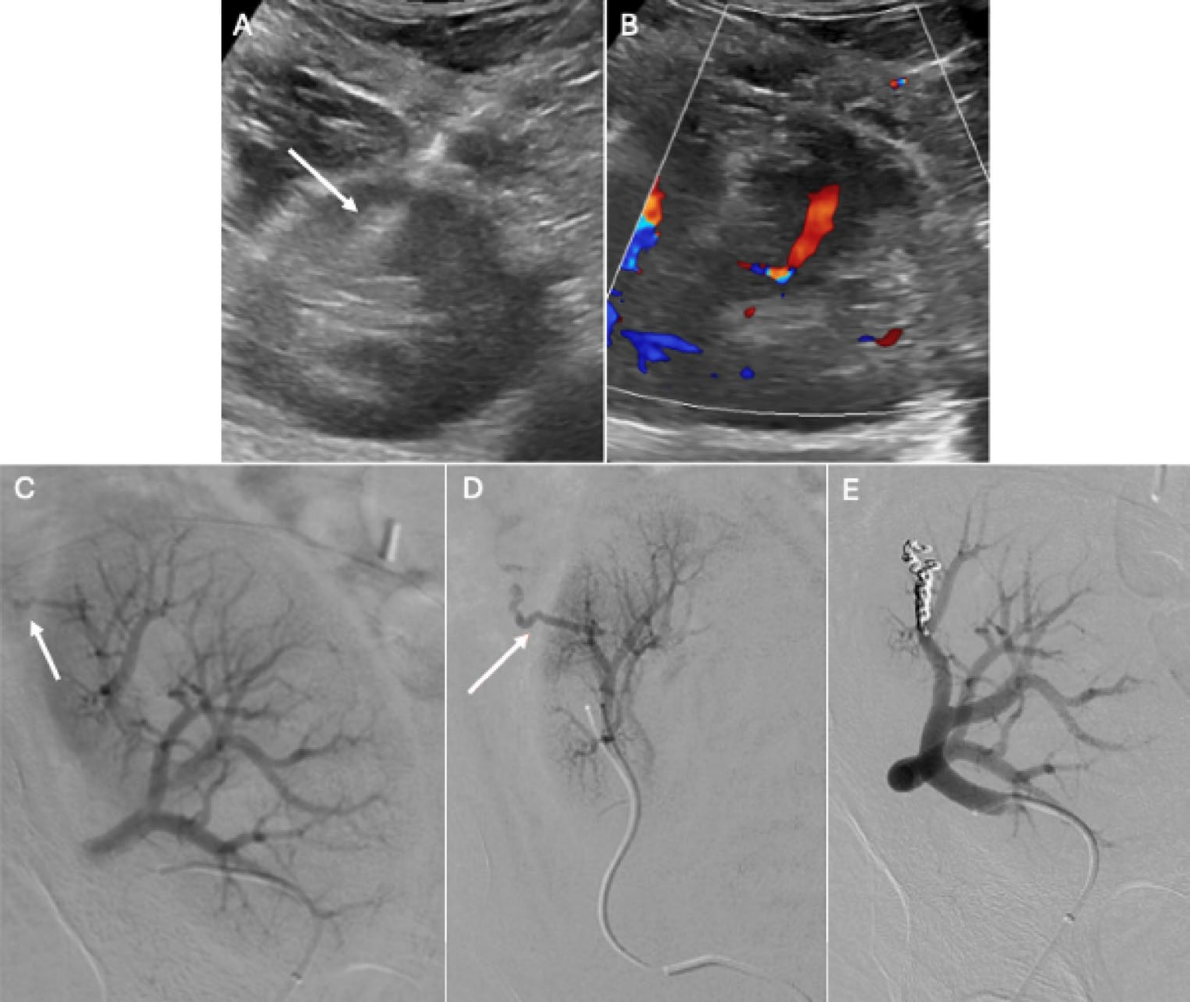
40-year-old male who presented for RLQ renal transplant biopsy due to concern for rejection. Ultrasound-guided percutaneous biopsy was performed with needle tip identified in the transplant renal cortex (A). Post-biopsy ultrasound (B) demonstrated a color Dopler jet external to the kidney, concerning for active arterial hemorrhage. Main renal artery (C) and upper pole renal artery (D) angiogram demonstrated active extravasation from a right upper pole renal artery branch. A super-selective right upper pole renal artery branch embolization was performed with Nester (Cook) 0.018’’ x 5 cm x 3 mm microcoils with good hemostasis (E)

Although historically performed blind, the procedure is now primarily done under image guidance, most commonly using US, but occasionally with CT if the kidney is poorly visualized to avoid unintentional arterial or venous injury [62]. During the procedure, the patient is placed supine. Via an anterior approach, a spring-loaded 16-gauge or 18-gauge needle is advanced under US-guidance into the renal cortex with the goal of collecting a minimum of 5–10 intact glomeruli for histologic diagnosis (Fig. 9) [24]. With image guidance, the procedure is very safe with a reported incidence of 0.2-2% of major complications [61, 63, 64]. A complete blood count (CBC) and coagulation studies should be obtained prior to renal biopsy as elevated INR and platelets < 120 × 10^3^ /µL are relative contraindications to the procedure [60]. Absolute indications include active kidney infection or active skin infection at the site of the biopsy [60]. Other imaging modalities to consider prior to biopsy include Doppler US, CT and MRI as they can help rule out external and structural causes of graft dysfunction.

#### Hemorrhage

Hemorrhage is the most common complication after renal transplant biopsy [65]. Most bleeding complications, such as transient hematuria and small hematomas, are minor and self-limiting [66, 67]. Larger hematomas may require treatment via open surgical or percutaneous drainage depending on their size and location or if symptomatic. Subcapsular hematomas specifically can lead to page kidney, which is characterized by declining renal function and acute-onset hypertension due to external compression of the renal parenchyma, requiring evacuation [68]. Bleeding complications can be visualized with Doppler, CT or MR. On non-contrasted CT, acute hemorrhage is hyperattenuating, decreasing in density over time due to clot lysis [69]. On MRI, signal intensity changes in a non-linear fashion depending on the age of the hemorrhage and stage of hemoglobin degradation [69]. Hematomas can be visualized on ultrasound as a heterogeneously echogenic collection, with decreasing echogenicity over time [69].

Major bleeding complications requiring additional management are significantly less common, with a reported incidence of 0.2–1.9% [64–67]. Treatment of major hemorrhage may involve hospitalization, transfusion of blood products as well as surgical or endovascular intervention, such as embolization, to achieve hemostasis (Fig. 9) (Table 1).

#### Arteriovenous fistula

**Fig. 10:**
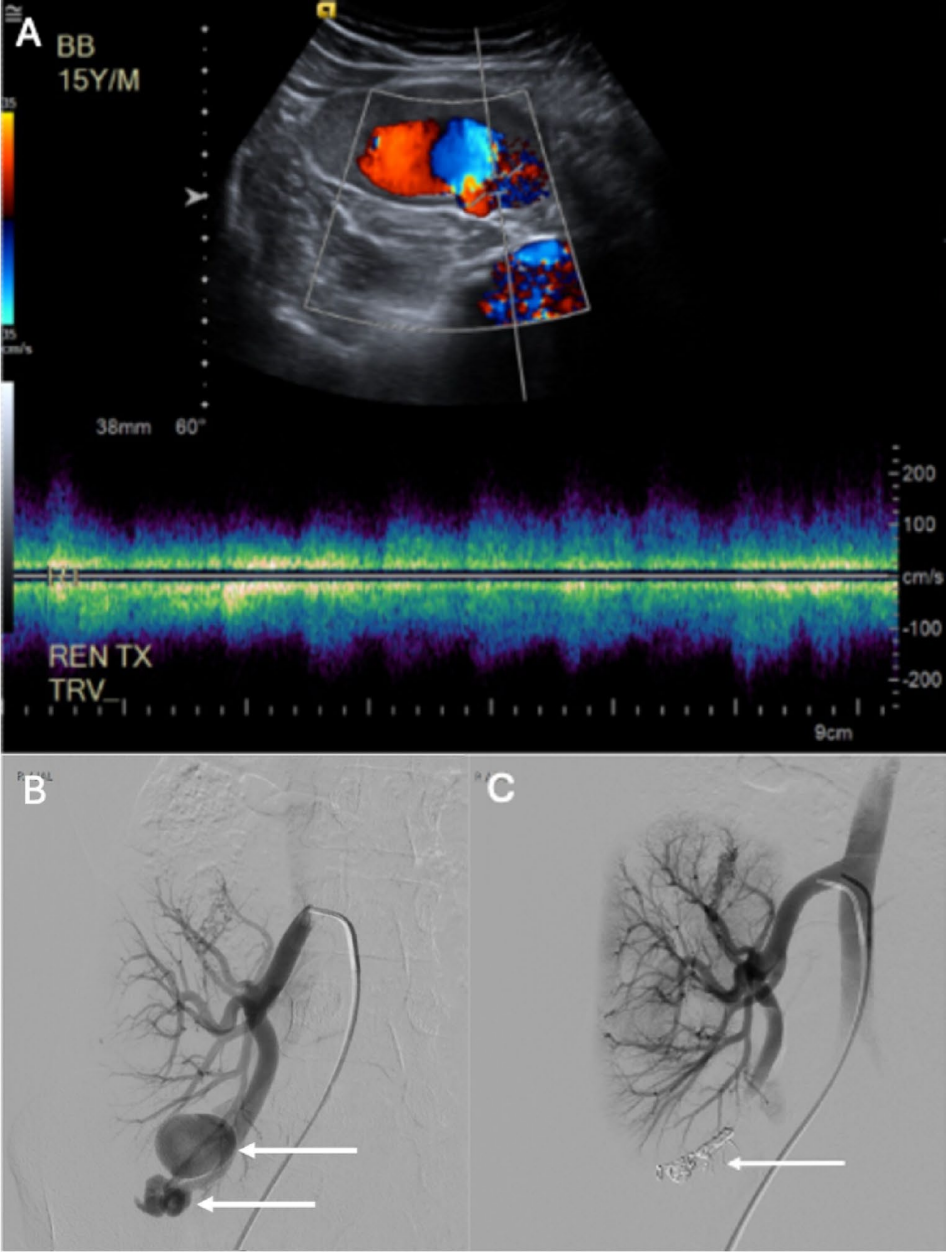
16-year-old male status post RLQ renal transplant who presented with hematuria following US-guided percutaneous renal transplant biopsy. Doppler US (A) and CT angiography (not pictured) confirmed an AVF with multiple pseudoaneurysms in the lower pole of the transplanted kidney. Selective transplant renal artery angiography demonstrated dilation of the lower pole segmental artery supplying the AVF with multiple pseudoaneurysms (white arrows) (B). Embolization was performed using 5 mm x 16 cm Azur hydrocoils (Terumo) followed by one vial of Onyx 18 liquid embolic (Medtronic). Post-embolization angiogram demonstrated complete resolution of the AVF and pseudoaneurysms (white arrow) with good opacification of the remaining renal vasculature (C)

Arteriovenous fistula (AVF) formation is a rare complication after graft biopsy [70]. Approximately 70% of the time, AVFs are asymptomatic and resolve spontaneously. Studies have reported a statistically significant increase in incidence observed with the use of a 14 gauge vs. 16 gauge needle [70]. If symptomatic, patients may present with unexplained hypertension, renal dysfunction and hematuria [63]. AVFs can be visualized directly on US as an abnormal connection between an artery and a vein with high-velocity flow. Other signs include color overlaying the surrounding soft tissue due to the intense vibration of the AVF, decreased arterial resistance, venous pulsatility or venous dilation due to increased blood flow [71]. Depending on the severity of symptoms, graft AVFs may be managed conservatively or successfully treated via transcatheter embolization (Fig. 10) (Table 1) [63, 72].

#### Pseudoaneurysm

**Fig. 11:**
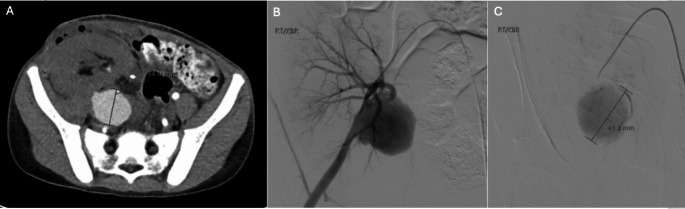
10-year-old female status post RLQ renal transplant who recently underwent US-guided percutaneous biopsy 2 weeks prior due to concern for rejection. Several days following biopsy, the patient developed RLQ pain and anemia, which required takeback to the operating room for evacuation of a perinephric hematoma. Repeat transplant US 10 days following surgery (not shown) demonstrated a suspected renal artery pseudoaneurysm, which was subsequently confirmed on CT angiography (A). Right eternal iliac angiogram confirmed the presence of a pseudoaneurysm arising from the inferior aspect of the transplant renal artery anastomosis, causing focal narrowing of the adjacent external iliac artery and sluggish inflow into the renal transplant artery (B and C). Given these findings, endovascular management was deferred, and the patient returned to the operating room for right external iliac artery reconstruction and transplant renal artery re-anastomosis

Although rare, pseudoaneurysm is a serious complication of graft biopsy that develops due to an iatrogenic arterial wall injury [58]. Symptoms include flank pain, hematuria, anemia and possible hemorrhagic shock. Patients may also have a bruit over the site of their graft [58]. The reported incidence of pseudoaneurysm development after biopsy is 5.6% [73]. Diagnosis is often incidentally discovered on routine imaging. While pseudoaneurysms appear cystic on ultrasound, color Doppler demonstrates characteristic blood flow in and out of the mass [74]. Pseudoaneurysms can also be visualized on CT, as a focal high attenuating lesion, similar in density to surrounding arteries [74]. Detection and treatment are important as rupture can lead to life-threatening hemorrhage [58, 75]. Depending on the size and location of the pseudoaneurysm, treatment may be managed endovascularly, most commonly with selective angioembolization, or with open surgery (Fig. 11) (Table 1) [58].

## Venous complications

Venous disruption, while less common than arterial, is a serious, graft-threatening complication post-renal transplant that requires immediate treatment to prevent graft loss. In this section, we will discuss common venous complications after renal transplant, namely transplant vein thrombosis as well as stenosis and thrombosis of native venous anatomy and review endovascular management options (Table 2).

**Table 2:** Summary of imaging findings of Post-Transplant venous complications

Venous Complications	Imaging Findings	Endovascular Treatment
Transplant vein		
*Thrombosis*	*US*: Lack of compressibility; reduced corticomedullary differentiation; absent venous color signal; bidirectional arterial flow *CTV/MRV*: Filling defect of the vein during venous phase; hypodense material in the lumen of the transplant vein *Angiography*: Decreased or failed opacification of the transplant vein	Thrombectomy +/- stent placement
Native vein		
*Stenosis*	*US*: Reduced diameter of the native vein *CTV/MRV*: Focal decrease in diameter of vein; increased collateral drainage depending on chronicity *Angiography*: Focal narrowing of native vein	Venoplasty +/- stent placement
*Thrombosis*	*US*: Lack of venous compressibility; absent venous color signal; visualization of thrombus within the vein *CTV*: Filling defect of the vein during venous phase; hypodense material in the lumen of the nativevein *Angiography*: Decreased or failed opacification of focal segment of the native vein	Thrombectomy +/ stent placement

### Transplant venous thrombosis

**Fig. 12:**
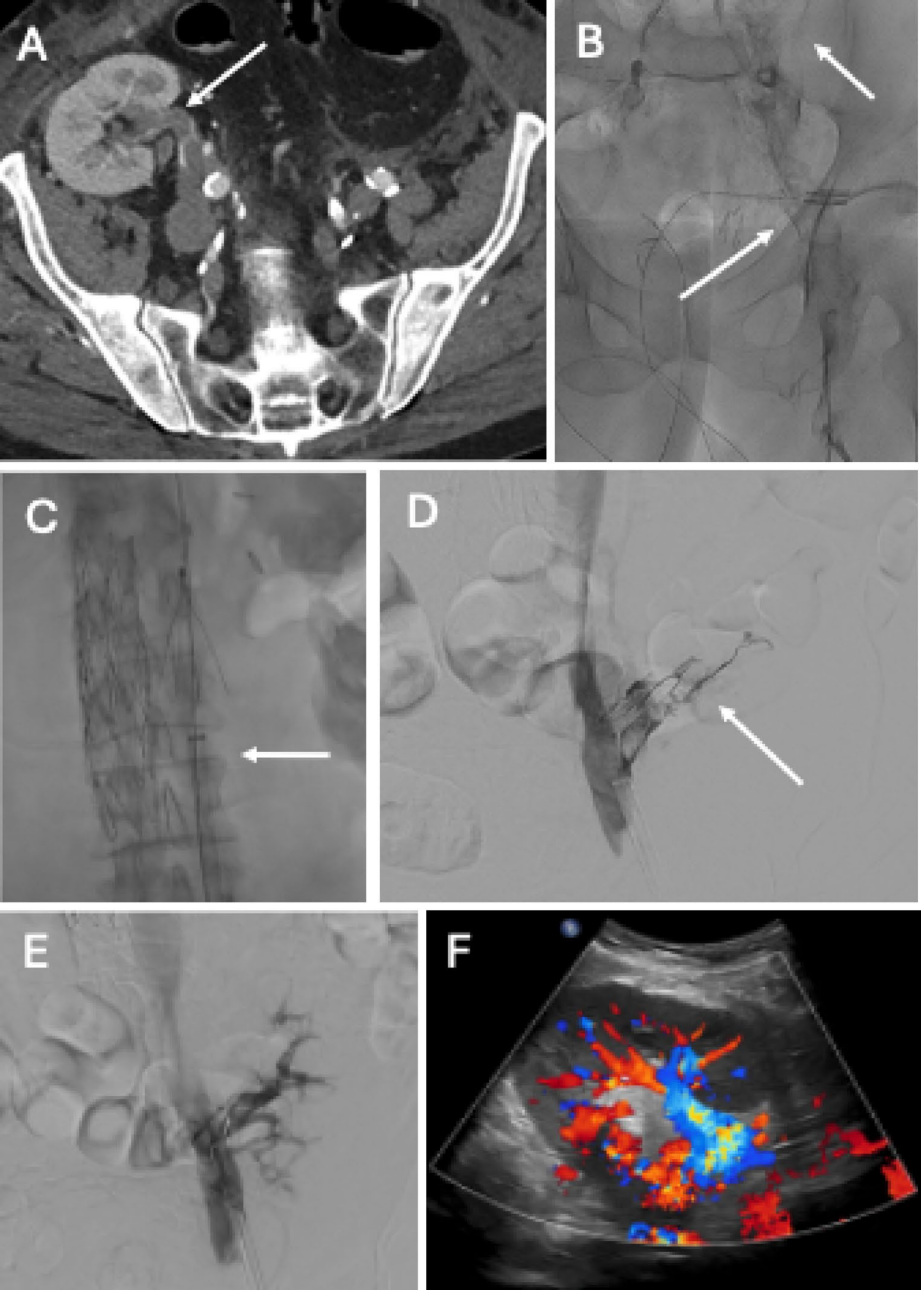
68-year-old male with remote history of RLQ renal transplant and lower extremity DVT requiring IVC filter, subsequently placed on warfarin, who was admitted for multicompartmental intracranial hemorrhage following ATV accident requiring warfarin discontinuation. The patient subsequently developed lower extremity swelling and acute renal failure. CT venogram demonstrated extensive bilateral lower extremity DVT extending into the transplant renal vein (A). Prone venogram performed from right saphenous venous access demonstrated extensive thrombus throughout the external iliac vein with non-opacification of the transplant renal vein (between white arrows) (B). Mechanical thrombectomy was performed using a 20 Fr Inari Flowtriever device (C). Subsequent venogram demonstrated markedly improved venous outflow with some persistent thrombus in the transplant renal vein (D). Aspiration and mechanical thrombectomy were performed in the transplant renal vein through a 5 French Vert catheter with repeat venogram demonstrating near resolution of thrombus with good outflow in the transplant renal vein (E). Renal US the following day demonstrated patent transplant vasculature (F)

Renal transplant vein thrombosis is an uncommon complication after renal transplant that nearly always results in graft failure [76, 77]. It has a reported incidence of 0.1- 1% [34–36, 38, 78, 79]. Clinical presentation includes pain refractory to medical management, oliguria, hematuria and signs of graft dysfunction [76, 80]. It most commonly occurs within the first two weeks post-transplant and is an important diagnosis to consider given the high rate of mortality from possible graft rupture or embolic complication [76, 80]. Risk factors for development of renal vein thrombosis include extended time on dialysis, history of previous thrombosis and right kidney transplantation [77]. Diagnosis can be made with renal Doppler US, CT angiography or MR angiography (Fig. 12) [77]. In addition to nonspecific signs, such as lack of compressibility and reduced corticomedullary differentiation, signs of transplant vein thrombosis include absent venous color signal and bidirectional arterial flow, the latter of which is pathognomonic for venous thrombosis. In the setting of transplant vein thrombosis, a positive arterial waveform can be seen during systole while reversal of the waveform is seen during diastole [81]. CTV or MRV would demonstrate a filling defect of the vein as well as hypodense material within the lumen of the vessel. Management is variable and depends on the extent of graft dysfunction. In cases of graft failure or necrosis, transplantectomy may be required [77, 80]. However, transplant salvage may be attempted with thrombolytic therapy, endovascular thrombectomy or open surgical thrombectomy (Fig. 12) [76].

### Complications of native vein

**Fig. 13:**
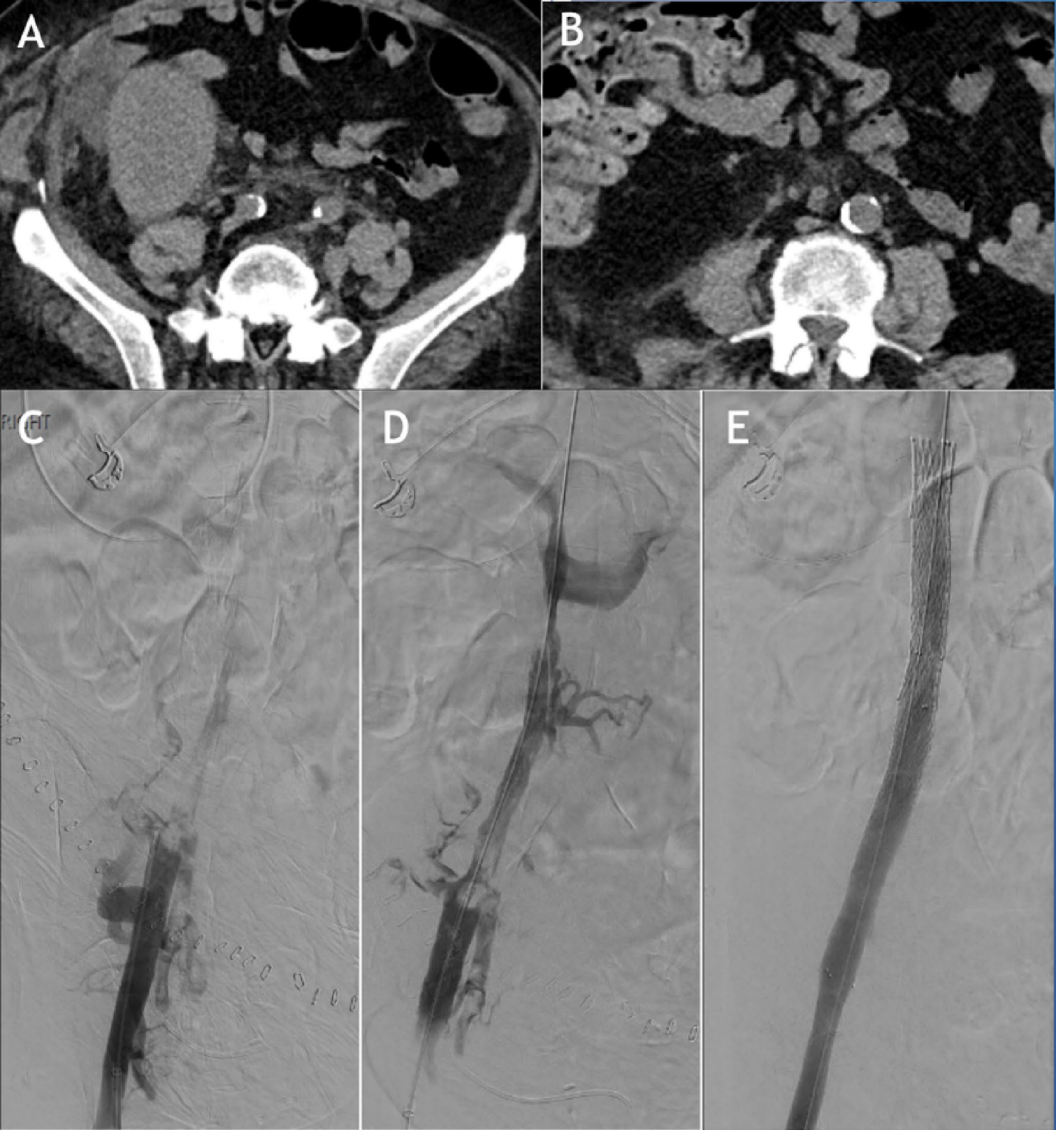
64-year-old female status post RLQ renal transplant admitted 1 week post-operatively with hypotension, hypothermia, acute renal failure and worsening right lower extremity swelling. Noncontrast CT demonstrated chronic occlusion/stenosis of the right common iliac vein, left common iliac vein and IVC with lumbar collaterals bypassing the areas of chronic central venous stenosis (A and B). Catheter venography demonstrated chronically occluded right external vein (upstream of the transplant venous anastomosis) as well as the right common iliac vein and IVC with extensive venous collateralization (C). The right external iliac vein and IVC were successfully recanalized using a small guidewire and Envoy catheter. Pull-back venogram (D) and intravascular US (not shown) demonstrated appropriate positioning within right common iliac vein and IVC. Venoplasty (not shown) was performed using 8 mm x 100 mm and 12 mm x 40 mm Conquest ballons (BD). Ultimately, a 16 × 80 mm Venovo stent (BD) was placed in the IVC as well as a 14 × 100 mm Venovo stent spanning from the right external iliac vein (above the level of the transplant renal vein inflow) to the IVC with 1 cm overlap with the first IVC stent. Completion venography demonstrated markedly improved flow through the right external iliac vein, right common iliac vein and IVC (E). At 1 and 6-month follow-up, patient reported resolution of right lower extremity swelling with GFR > 60 and patent renal and pelvic veins on Doppler and CT venography (not shown)

Iliac vein complications are incredibly rare post-transplant, with only a handful of cases documented in the literature [82]. Stenosis and deep vein thrombosis are two potential complications that can impair venous outflow and result in graft dysfunction (Fig. 13) [82]. Native vein injury may develop as a result of May-Thurner syndrome, external compression from the graft or clamp-related trauma [76]. Occlusion of the native vein can also develop secondary to preexisting venous stenosis [82]. Anticoagulation is appropriate initial management if the thrombus spares the transplant vein followed by more invasive intervention, namely thrombectomy and venoplasty with or without stent placement, if medical management fails (Fig. 13) [83].

## Lymphatic complications

Lymphatic complications are well-documented after renal transplant with a reported incidence ranging between 0.6–22% [84]. They primarily develop due to disruption of the extensive lymphatic drainage pathways that exist around the iliac vessels or through incomplete ligation of lymphatic vessels removed with the donor kidney [85]. Two common sequelae of lymphatic disruption are lymphorrhagia - extravasation of lymph into the surrounding region - and lymphocele - a lymph-filled collection without an epithelial lining. Lymphoceles most commonly develop between 2 weeks and 6 months following transplant, with a peak incidence around 6 weeks [85,86]. Previous literature has outlined several treatment factors associated with higher rates of lymphocele development including the use of heparin, high-dose corticosteroids, early patient mobilization and retroperitoneal transplantation as opposed to intraperitoneal [84,86,87]. Moreover, studies have observed higher rates of lymphocele formation with laparoscopic removal of the donor kidney, with some authors advocating for more extensive back-table preparation for kidneys extracted in this manner [84].

**Fig. 14:**
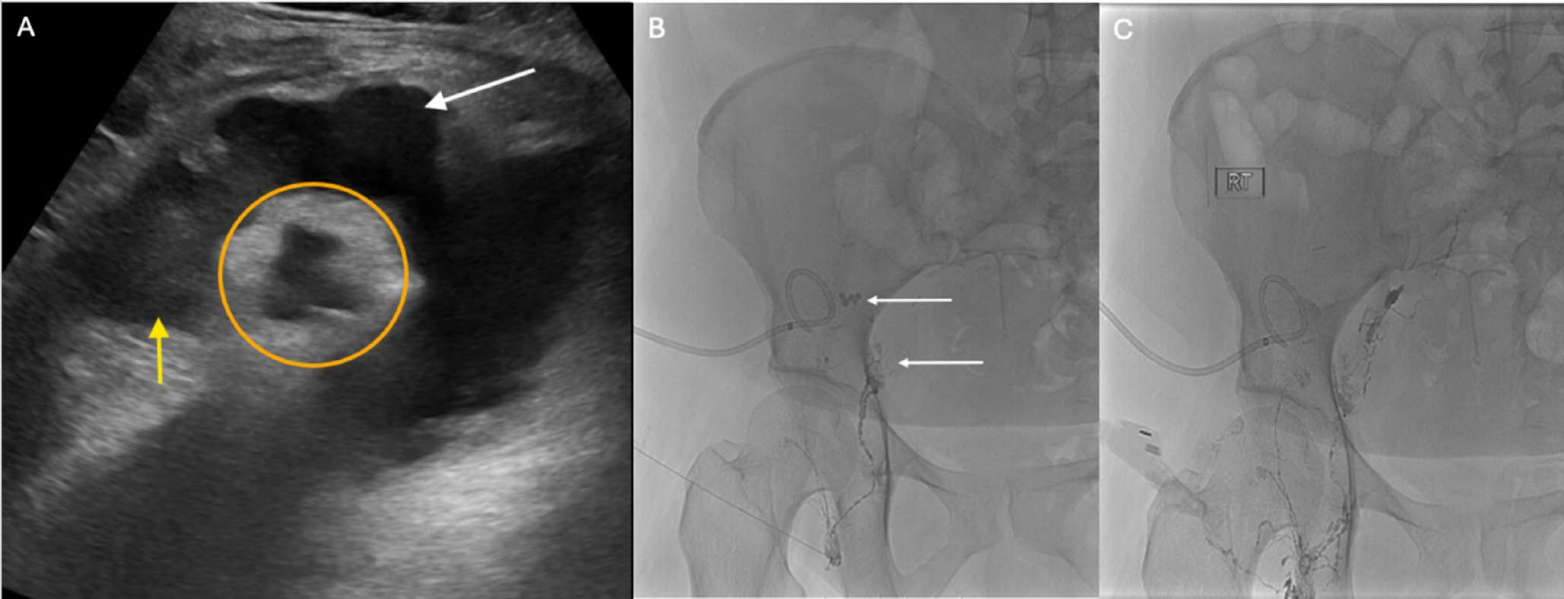
40-year-old female status post RLQ renal transplant readmitted two months after transplant with increasing shortness of breath, weight gain and lower extremity DVT. A perinephric fluid collection (white arrow) was identified on admission renal US (A; yellow arrow– renal parenchyma; orange circle– renal hilum). Subsequently, a percutaneous drain was placed using ultrasound-guidance (not shown). Drain output was consistently 400-500 cc per day of thin, light fluid. Drainage fluid labs including creatinine, triglycerides, cell count and culture were unrevealing. Lymphangiogram with lipiodol was performed via a right inguinal lymph node to assess for possible lymphatic leak, which demonstrated contrast pooling into the region of the perinephric collection and drain (B). Lymphatic embolization was performed with approximately 1 cc of 1:7 n-BCA-Lipiodol mixture. Post-embolization imaging demonstrated no further communication (C). Lymphocele sclerotherapy was performed through the percutaneous drain with 7 cc of doxycycline for 90 min following embolization

Diagnosis of lymphatic leak or lymphocele can be aided by both imaging and laboratory findings. Since they are often asymptomatic, lymphatic complications are commonly found incidentally on routine imaging [86]. Ultrasound is a safe, effective initial imaging modality to visualize the fluid collection and determine its location in relation to the transplant (Fig. 14). CT and lymphangiography can also be used for diagnostic evaluation, the latter of which also serves as a form of percutaneous treatment (Fig. 14) [85, 88]. Lymphangiography involves US-guided administration of ethiodized oil, Lipiodol, into a lymph node, commonly in the inguinal region. The lipiodol allows for visualization of any abnormalities or leaks as it travels through the lymphatic system. It also provides a slight therapeutic benefit that has demonstrated occasional success in leak management without the need for administration of additional embolic materials [89]. Biochemical analysis of any drained or aspirated fluid allows differentiation of lymphatic leak from other possible sources, including urinoma or superinfected collection. Relevant values that are obtained from the sampled fluid include creatinine, potassium, culture/gram stain and cell count [90]. As opposed to urinomas, lymphoceles will demonstrate creatinine and potassium levels similar to serum levels and a high lymphocyte count [90].

Imaging and timing can also be helpful tools in differentiating between the various types of collections. Lymphoceles and urinomas both appear anechoic on US, similar to simple fluid [68, 91] and on non-contrasted CT will appear as a hypoattenuating collection. On MR, they appear T1 hypointense and T2 hyperintense. However, unique to urinomas, on a contrasted study, a urinoma will fill with contrasted material due to their connection to the urinary system [91]. As well, urinomas commonly appear early in the post-operative period as they are typically the result of iatrogenic trauma to the organ, incomplete anastomosis or ischemic necrosis due to vascular compromise while lymphoceles usually present about 4–8 weeks post-operatively [92]. However, if other testing is inconclusive and there is high suspicion, renal dynamic scintigraphy is a highly sensitive diagnostic tool for urinoma that can be used for confirmation [92]. Abscess formation is more variable in timing as they can arise as either a surgical complication or a secondary infection of a fluid collection such as a urinoma or hematoma. On US, abscesses appear as complex collections with heterogenous echogenicity and on CT they are peripherally enhancing due to increased blood flow and inflammation in the region [68].

**Fig. 15:**
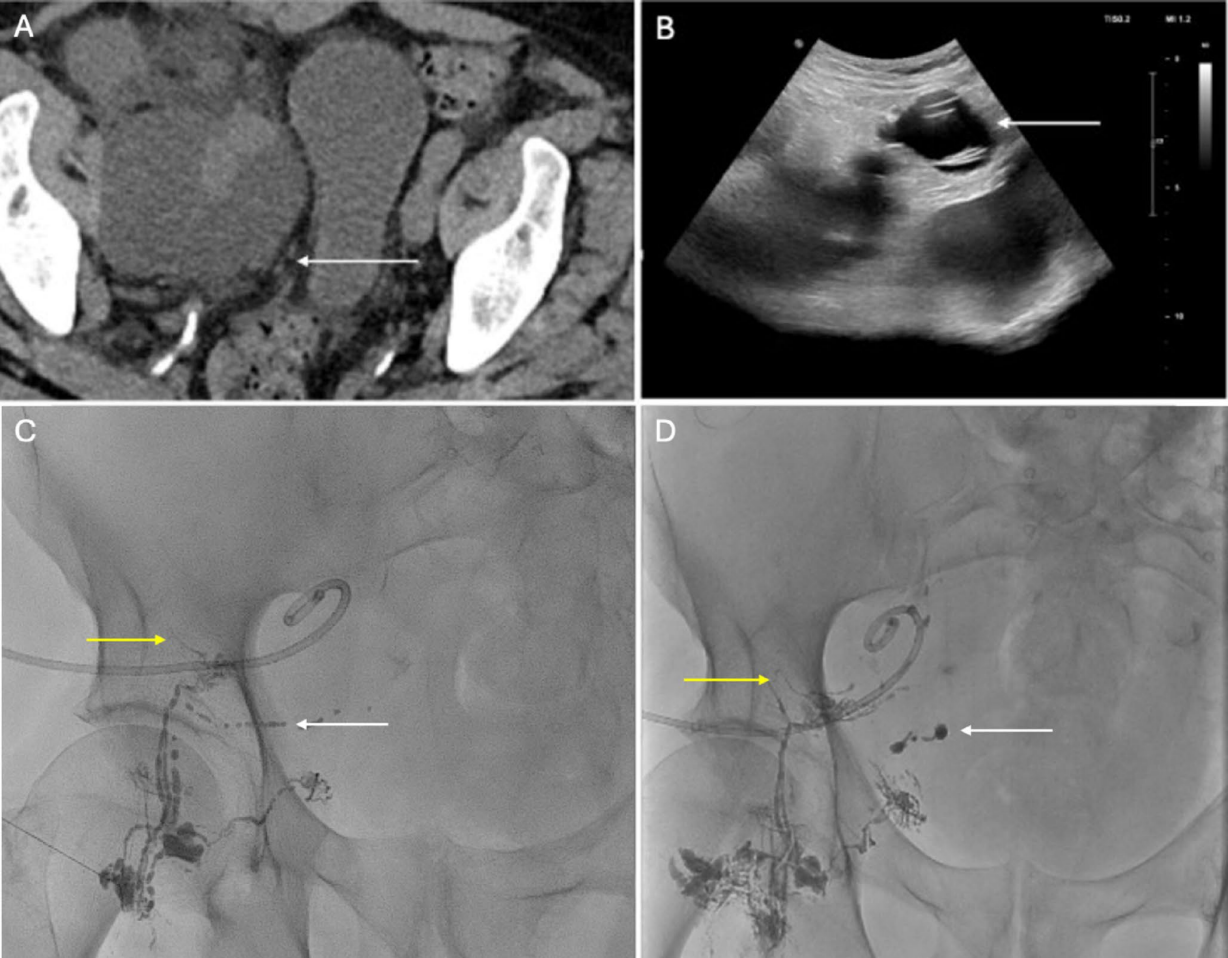
72-year-old male status post RLQ renal transplant one month prior with significant output through the surgical drain concerning for lymphatic leak. Admission non-contrast CT revealed a low-density fluid collection (white arrow) in the right hemipelvis inferior to the RLQ renal transplant, suspicious for lymphocele (A). A percutaneous drain was placed under US-guidance into the pelvic collection (white arrow) (B). Lymphangiogram with lipiodol performed via a right inguinal lymph node demonstrated dilated and tortuous lymphatic channels (yellow arrow– lateral lymphatic channel; white arrow– medial lymphatic channel) throughout the right hemipelvis, with lipiodol pooling in the collection near the site of the drain (C). Antegrade embolization with 5:1 mixture of lipiodol: n-BCA glue was then performed, resulting in good embolic opacification of the lymphatic channels at the site of the leak (D)

Although a common occurrence, only a small minority of lymphatic complications following renal transplant require treatment as most resolve spontaneously [86]. If treatment is required due to symptoms or mass effect, initial management usually involves minimally invasive options such as percutaneous drainage or aspiration, with or without sclerotherapy (Fig. 14) (Table 3) [93]. Sclerotherapy agents include fibrin glue, sodium tetradecyl sulphate (STS) or tetracycline antibiotics [86, 94]. Studies have also demonstrated successful management of persistent lymphatic leaks with image-guided embolization [95] (Figs. 14 and 15) or transperitoneal balloon-fenestration to facilitate drainage into the peritoneal cavity (Table 3)[96]. Laparoscopic or open surgical management may also be performed for patients that fail less aggressive treatment.^86^.

**Table 3:** Summary of imaging findings of Post-Transplant lymphatic complications

Lymphatic Complications	Imaging Findings	Treatment
*Lymphatic Leak*	*US*: Free fluid *CT/MR Lymphangiography*: Free fluid; extravasation from lymphatic ducts *Lymphangiogram*: Dilated, tortuous lymphatic channels with extravasation	Lymphangiogram; embolization; trans-peritoneal balloon fenestration
*Lymphocele*	*US*: Anechoic collection, resembling simple fluid *CT*: Hypoattenuating collection *MR*: Fluid collection that is hypointense on T1 and hyperintense on T2 *Lymphangiogram*: Lipiodol pooling	Percutaneous drainage/aspiration; sclerotherapy

## Conclusion

Renal transplantation is a life-saving procedure for patients with end stage renal disease, but it is not without potential pitfalls. Vascular and lymphatic complications have the potential to result in graft failure if not identified early. Abdominal radiologists should be able to identify these complications and understand the available management strategies to ensure continued graft preservation for patients.

## Data Availability

No datasets were generated or analysed during the current study.
